# Automated *Mycoplasma genitalium* molecular macrolide resistance detection and nucleic acid target semi-quantitation: patient demographic considerations

**DOI:** 10.1128/spectrum.00738-25

**Published:** 2025-05-22

**Authors:** Josephine Moore, Trinity Krueger, Amanda Zapp, Stephen C. Lavey, Kimber L. Munson, Irene A. Stafford, Michael E. Newcomb, Brian Mustanski, Erik Munson

**Affiliations:** 1 Department of Medical Laboratory Science, Marquette University 5505, Milwaukee, Wisconsin, USA; 2 Loyola University Parkinson School of Health Sciences and Public Health, Maywood, Illinois, USA; 3 Department of Obstetrics and Gynecology, McGovern Medical School at UTHealth, Houston, Texas, USA; 4 Division of Maternal Fetal Medicine, McGovern Medical School at UTHealth, Houston, Texas, USA; 5 Division of Infectious Diseases, Northwestern University 273158 , Chicago, Illinois, USA; 6 Department of Medicine, Northwestern University 3270, Evanston, Illinois, USA; 7 Feinberg School of Medicine, Northwestern University 3270, Evanston, Illinois, USA; 8 Institute for Sexual and Gender Minority Health and Wellbeing, Northwestern University 205058, Chicago, Illinois, USA; 9 Department of Medical Social Services, Northwestern University 205058, Chicago, Illinois, USA; 10 Wisconsin Clinical Laboratory Network Laboratory Technical Advisory Group, Madison, Wisconsin, USA; Houston Methodist Hospital, Houston, Texas, USA

**Keywords:** *Mycoplasmoides genitalium*, transcription-mediated amplification, macrolide resistance, semi-quantitation

## Abstract

Recent work has optimized parameters of a real-time reverse transcriptase PCR-based laboratory-developed test on the Panther Fusion system which detects Mycoplasma genitalium-specific macrolide resistance-associated mutations LDT (MRM-LDT) from primary swab and urine specimens. In this study, MRM-LDT was applied to a large multi-demographic study set to further characterize *M. genitalium* resistance in the United States. A total of 2,145 primary clinical specimens testing positive for *M. genitalium* 16S rRNA by transcription-mediated amplification (TMA) were initially titered by the same assay using serial 10-fold dilutions to determine relative target nucleic acid burden. Specimens were then processed for MRM-LDT. Findings were stratified by men who have sex with men (MSM; *n* = 3 settings), community care (*n* = 2), and university student (*n* = 3) populations. The mean log_10_ target nucleic acid titer of a TMA-positive specimen was 3.46 (median 3; range 0–10). A 43.2% MRM-LDT detection rate was found in specimens derived from community care settings. Analogous assessments of university student and MSM demographics revealed 60.8% and 62.0% detection, respectively (*P* ≤ 0.0004 versus community care). Within the university student demographic, differences existed in target nucleic acid titer for two settings (*P* = 0.01); similar differences were encountered between a local MSM cohort and two that were nationally based (*P* ≤ 0.01). Within the university cohort, MRM-LDT detection rate was increased in symptomatic patients (*P* = 0.005). *M. genitalium* macrolide resistance rates among multiple demographics, as determined by MRM-LDT, are high in the United States and are consistent with target nucleic acid burden within the primary specimen. However, caveats experienced within subgroupings of these demographics support reflex MRM-LDT on *M. genitalium*-positive specimens.

**IMPORTANCE:**

Data generated from a high-throughput, automated system and presented in this report expand upon knowledge of *Mycoplasma genitalium*-specific macrolide resistance in the United States and may further inform providers on population- or demographic-based considerations for macrolide resistance mutation determination in *M. genitalium*.

## INTRODUCTION

Beyond improvements in laboratory detection of *Mycoplasma genitalium* (1, 2), including FDA-cleared commercial nucleic acid amplification assays (3–5), providers are now confronted with the possibility of *M. genitalium* treatment failure relative to first-line macrolide therapeutic agents (6–8). In recognition of this paradigm, the US Centers for Disease Control and Prevention (CDC) now predicates initial therapeutic strategies for *M. genitalium* infection based on availability of a laboratory result for macrolide resistance (9). While culture and antimicrobial susceptibility testing systems exist for *M. genitalium* and have contributed to the understanding of alternative therapeutic agents (10, 11), turnaround times for this traditionally slow-growing bacterium may not be optimal for clinical practice (12). As such, molecular assays for detection of point mutations correlating with macrolide treatment failure have been developed in research (13, 14) and diagnostic laboratory (15, 16) settings. However, to date, these assays have neither received clearance from the US Food and Drug Administration nor are available for widespread use in the United States.

Our group recently optimized parameters for a real-time reverse transcriptase PCR-based *M. genitalium*-specific macrolide resistance-associated mutations (MRM) laboratory-developed test (LDT) that is facilitated by commercial automation (17). Detection of MRM by the MRM-LDT occurred at a 91% concordance rate with reference Sanger sequencing. With the prospect of a modality that could support high-throughput testing, this investigation sought to chronicle MRM incidence in US patient settings using a large primary clinical specimen set that initially yielded a positive transcription-mediated amplification (TMA) test result for *M. genitalium*.

## MATERIALS AND METHODS

### Primary clinical specimens

Aliquots of first-void urine were added to Aptima urine specimen transport tubes (Hologic, Inc., San Diego, CA, USA) per package insert protocol within 24 hours following specimen procurement. Primary genital and self-collected rectal swab specimens were delivered to Aptima unisex or Aptima multitest swab specimen collection kits. All aliquots or collected swab specimens were maintained at 2°C–30°C prior to test performance and tested within 30 days of collection.

### Study settings

In this investigation, three midwestern US college settings contributed primary specimens to this assessment. Incidence of *M. genitalium* nucleic acid detection at two of these institutions has been reported at 7% (18), while prevalence and therapeutic intervention failure are the subject of a second report (19). A second category of primary specimen collection was comprised of two community care settings (one in midwestern, one in southern United States). Reports have characterized *M. genitalium* detection at approximately 6%–11% in females and 7% in males within these locales (20–22). Historic high-prevalence sexually transmitted infection (STI) communities (23, 24) fed into the first two study setting categories. A third category of primary specimen collection focused on cohorts of young men who have sex with men (MSM), both in local (MSM setting 1) (25) and national (MSM settings 2 and 3) scope. STI detection rates in MSM setting 1 have been published (26), with *Chlamydia trachomatis* nucleic acid detection rates of 1.4% and 8.2% in urine and rectal swab specimens, respectively. Analogous values for *M. genitalium* were 9.1% and 21.5%. This investigation was approved by the Marquette University Institutional Review Board.

### Commercial *M. genitalium* TMA

Specimens were previously screened for *M. genitalium* 16S rRNA by TMA-based Aptima Mycoplasma genitalium Assay (Hologic) using Panther automation (Hologic). Testing was performed per package insert indications for urine and genital swab specimens. Off-label testing on rectal swab specimens was procured as an LDT using the same assay, as reported previously (26, 27).

### *M. genitalium* 16S rRNA semi-quantitation

Primary specimens yielding detectable 16S rRNA by the Aptima Mycoplasma genitalium Assay were serially diluted and subjected to the same assay for titer determination. Briefly, 332 and 222µL volumes of remnant swab tubes or urine aliquot tubes, respectively, were delivered to fresh Aptima multitest swab specimen collection (containing 2.9 mL transport lysis medium) or Aptima Urine Specimen Transport Tubes (containing 2.0 mL transport lysis medium) where appropriate. In a similar fashion, diluted material was subjected to additional 1:10 dilutions in respective media until a titer was determined (defined as the reciprocal of the highest dilution to yield a detectable result with the Aptima Mycoplasma genitalium Assay). Titer values are expressed as log_10_ values throughout this report.

### *M. genitalium* MRM-LDT using ASR

A real-time reverse-transcriptase PCR-based LDT detecting point mutations A2058C, A2058G, A2058T, A2059C, and A2059G (*Escherichia coli* numbering), performed on the Panther Fusion System (Hologic), was previously described in detail (17). To summarize, primer/probe reconstitution (PPR) mix was comprised of 0.6 µM proprietary *M. genitalium* MRM analyte-specific reagents (ASR) primer/0.4 µM *M*. *genitalium* macrolide MRM ASR probe oligonucleotides, 0.6 µM internal control ASR primer/0.4 µM internal control ASR detection probe oligonucleotides, 2 mM MgCl_2_, and 60 mM KCl. All PPR reagents were provided by Hologic. General-purpose reagent cartridges containing lyophilized nucleotide bases, reverse transcriptase, and DNA polymerase (Hologic) were used with the PPR mix. Data output reports the detection of *M. genitalium* MRM but does not specify genotype.

Three hundred microliter aliquots of residual swab specimens with TMA-detectable *M. genitalium* RNA were delivered to clean, conical bottom specimen tubes into which a 300 µL aliquot of specimen transport medium mixed with proprietary Panther Fusion Open Access diluent additive (in a 100:1 vol/vol ratio) was dispensed. Six hundred-microliter aliquots of residual urine specimens that were TMA-positive for *M. genitalium* RNA were added to clean, conical bottom specimen tubes into which 3.75 µL of the proprietary Panther Fusion Open Access diluent additive was dispensed.

Following direct tube sampling and target capture-based nucleic acid extraction, amplification parameters included an initial reverse transcription step of 46°C for 8 min, 95°C for 2 min, followed by 45 cycles of 95°C for 5 sec and 60°C for 22 sec. These parameters, along with FAM and Quasar 705 fluorescence channel thresholds of 1,000 units and a default baseline correction slope limit of 250 units, were incorporated into an open-access LDT protocol.

### Data analysis

Aptima Mycoplasma genitalium Assay results were instrument-defined and qualitative in nature. MRM-LDT cycle threshold (*C_T_*) values < 40 were considered positive for detection of *M. genitalium* MRM. The significance test of proportions determined if differences in either proportions of log_10_ titer distribution or MRM-LDT detection rate were significant. The *t* test for independent samples determined if differences in log_10_ titer frequency distribution were significant as a function of patient location. The alpha level was set *a priori* at 0.05 before the investigations commenced, and all *P* values are two-tailed.

## RESULTS

### Initial findings

In the context of this investigation, 2,416 primary clinical urogenital and extragenital specimens were gathered. A total of 266 of the specimens initially yielded non-detectable results via commercial *M. genitalium* TMA and were subsequently used to verify specificity of the MRM-LDT; all testing within this subset of specimens generated negative results for MRM, with the exception of one that generated a non-valid result due to absence of internal control amplification. Within 2,150 specimens yielding a result of detected via commercial *M. genitalium* TMA, 2,145 generated results by MRM-LDT (two specimens had insufficient residual volume for analysis; three specimens yielded internal control amplification failure). Of all evaluable specimens, the MRM-LDT non-valid rate was 0.17% (4/2414).

### Semi-quantitation of *M. genitalium* rRNA target burden in primary clinical specimens screened positive by commercial TMA and correlation with MRM result determined by MRM-LDT

Primary specimens screening positive for *M. genitalium* by commercial TMA were gathered for log_10_ *M. genitalium* TMA titer determination of *M. genitalium*-specific 16S rRNA. A median log_10_ TMA titer of 3 was calculated within the 2,145 evaluated specimens, with a mean log_10_ *M. genitalium* TMA titer of 3.46 (equating to *M. genitalium*-specific rRNA being detected at a 1:2,900 dilution of the average primary clinical specimen; data not illustrated). Log_10_ *M. genitalium* TMA titer values were evenly distributed between the 1:1 and 1:1,000,000 dilutions of individual primary specimens (Table 1).

**TABLE 1:** log_10_ *M. genitalium* TMA titer distribution and MRM-LDT detection rates derived from 2,145 specimens collected from university, community, and MSM settings that initially yielded detectable *M. genitalium* RNA by commercial TMA

Log_10_ *M. genitalium* TMA titer	Number (% of total) of specimens	Number (% within log_10_ *M. genitalium* TMA titer subset) with detectable *M. genitalium* macrolide resistance by MRM-LDT
0	208 (9.7)	2 (1.0)
1	232 (10.8)	17 (7.3)
2	314 (14.6)	83 (26.4)
3	340 (15.9)	218 (64.1)
4	338 (15.8)	290 (85.8)
5	312 (14.5)	270 (86.5)
6	241 (11.2)	218 (90.5)
7	114 (5.3)	100 (87.7)
8	39 (1.8)	37 (94.8)
9	6 (0.3)	6 (100.0)
10	1 (0.05)	1 (100.0)

A total of 1,094 specimens (51.0%) yielded a log_10_ *M. genitalium* TMA titer value lower than the mean value (Table 1). The MRM-LDT detection rate was increased in specimens yielding a log_10_ *M. genitalium* TMA titer ≥4 (87.7%) when compared to specimens with a log_10_ *M. genitalium* TMA titer ≤3 (29.3%; *P* < 0.0002).

### *M. genitalium* target rRNA burden semi-quantitation and MRM detection, stratified by patient setting

*M. genitalium* MRMs were detected by MRM-LDT in 1,242 (57.9%) of *M. genitalium* TMA-positive specimens assayed (Table 2). Resistance rates in both the cumulative university and MSM settings (≥60.8%) were increased over those in the community setting (43.2%; *P* ≤ 0.0004). Median log_10_ *M. genitalium* TMA titers were also increased in both the combined MSM and university settings when compared to the community setting. Mean log_10_ *M. genitalium* TMA titer data were also increased in the MSM (3.62) and university (3.78) settings when compared to community care (2.79; both *P* < 0.0001).

**TABLE 2:** Semi-quantitative *M. genitalium* RNA detection and MRM-LDT detection from primary clinical specimens screening positive for *M. genitalium* by commercial TMA and emanating from university, community, and MSM clinical settings

Specimen source	N	Median log_10_ *M. genitalium* TMA titer	Mean log_10_ *M. genitalium* TMA titer	Number (%) with detectable *M. genitalium* macrolide resistance by MRM-LDT
University setting 1	88	4	4.07	55 (62.5)
University setting 2	37	3	3.11	19 (51.4)
University setting 3	5	3	3.80	5 (100.0)
All university testing	130	4	3.78	79 (60.8)
Community setting 1	402	3	2.80	175 (43.5)
Community setting 2	56	3	2.71	23 (41.1)
All community testing	458	3	2.79	198 (43.2)
MSM setting 1	720	3	3.43	441 (61.3)
MSM setting 2	615	4	3.73	380 (61.8)
MSM setting 3	222	4	3.96	144 (64.9)
All MSM testing	1557	4	3.62	965 (62.0)
Cumulative results	2145	3	3.46	1242 (57.9)

### University setting

**Fig 1:**
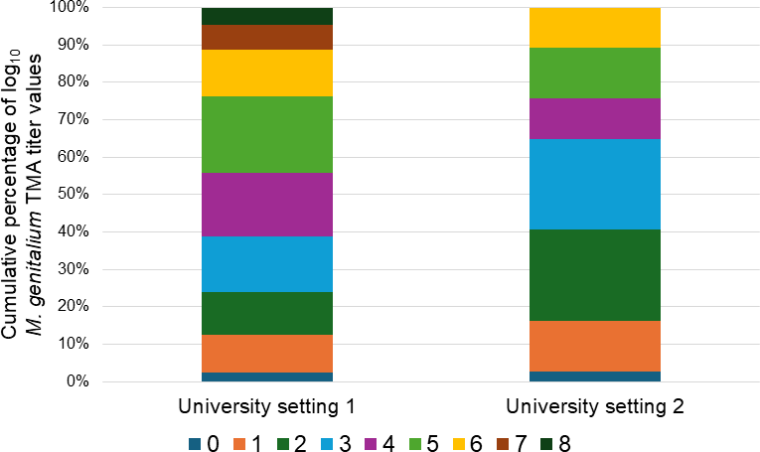
Comparative distribution of semi-quantitative *M. genitalium* RNA determination values from primary clinical specimens initially screening positive for *M. genitalium* by commercial TMA and derived from university setting 1 and university setting 2.

Data from university setting 3 were excluded from further analysis due to a low *n* value of *M. genitalium*-positive screens. An increased MRM-LDT detection rate was noted within a limited number of specimens from university setting 1 (62.5%) when compared to university setting 2 (51.4%), though the difference was not significant (*P* = 0.25). However, median and mean log_10_ *M. genitalium* TMA titer distributions (Table 2) between the two settings suggest (organism burden) differences in the testing populations (*P* = 0.01 for mean log_10_ *M. genitalium* TMA titer). Furthermore, well over 50% of the log_10_ *M. genitalium* TMA titer values from university setting 2 were ≤3 (Fig. 1). In contrast, nearly 50% of log_10_ *M. genitalium* TMA titer values from university setting 1 were ≥5.

Data regarding patient clinical status were available from university settings 1 and 2. Symptomatic rates in these settings ranged from 43.2% to 53.4% (*P* = 0.30). When comparing university settings 1 and 2, mean log_10_ *M. genitalium* TMA titer (*P* = 0.26), median log_10_ *M. genitalium* TMA titer, and MRM-LDT detection (*P* = 0.78) data from symptomatic patients were consistent (Table 3). Cumulative values of the aforementioned analytes, including a 71.4% MRM-LDT rate, were increased in university symptomatic patients (*P* ≤ 0.036). In asymptomatic patients, both mean and median log_10_ *M. genitalium* TMA titer data from university setting 1 demonstrated increases over those of university setting 2 (*P* = 0.03 for mean log_10_ titer data; Table 3). However, these differences did not translate into significant differences in MRM-LDT rates (*P* = 0.33).

**TABLE 3:** Semi-quantitative *M. genitalium* RNA detection and MRM-LDT detection from primary clinical specimens screening positive for *M. genitalium* by commercial TMA and emanating from university settings, stratified by symptomatic status

Parameter	Symptomatic				Asymptomatic			
	University setting 1	University setting 2	*P* value between settings	Cumulative	University setting 1	University setting 2	*P* value between settings	Cumulative
Specimens positive by *M. genitalium* TMA	47	16		63	41	21	–^*c*^	62
Median log_10_ *M. genitalium* TMA titer	4	3.5		4	4	2	–	3
Mean log_10_ *M. genitalium* TMA titer	4.30	3.69	0.26	4.14^*a*^	3.80	2.67	0.03	3.42
Number (%) with detectable *M. genitalium* macrolide resistance by MRM-LDT	34 (72.3)	11 (68.8)	0.78	45 (71.4)^*b*^	21 (51.2)	8 (38.1)	0.33	29 (46.7)

^*a*^ *P* = 0.036 versus rate in asymptomatic.

^*b*^ *P* = 0.005 versus rate in asymptomatic.

^*c*^ “–” indicates not applicable.

### Community setting

Percentage MRM-LDT detection rate (*P* = 0.73), mean log_10_ *M. genitalium* TMA titer (*P* = 0.72), and median log_10_ *M. genitalium* TMA titer data were consistent among the two community settings (Table 2). When MRM-LDT detection rates for the two community locations were stratified by log_10_ *M. genitalium* TMA titer findings, community setting 2 yielded lower comparative MRM-LDT detection rates for high-TMA titer specimens (Table 4).

**TABLE 4:** *M. genitalium* MRM-LDT detection rates from primary clinical specimens positive for the agent, stratified by community setting location and antecedent semi-quantitation of *M. genitalium* target RNA burden by commercial TMA

log_10_ *M. genitalium* TMA titer	Community setting 1		Community setting 2	
	N	Number (%) with detectable *M. genitalium* macrolide resistance by MRM-LDT	N	Number (%) with detectable *M. genitalium* macrolide resistance by MRM-LDT
0	39	0 (0.0)	7	0 (0.0)
1	56	7 (12.5)	9	0 (0.0)
2	92	19 (20.7)	9	3 (33.3)
3	86	42 (48.8)	9	5 (55.6)
4	56	49 (87.5)	15	11 (73.3)
5	43	35 (81.4)	4	2 (50.0)
6	23	17 (73.9)	3	2 (66.7)
7	4	3 (75.0)	–^*a*^	–
8	3	3 (100.0)	–	–

^*a*^ “–” indicates no specimens were encountered that had log10 *M. genitalium* TMA titers of 7 or 8; thus, the MRM-LDT could not be performed.

### MSM setting

**Fig 2:**
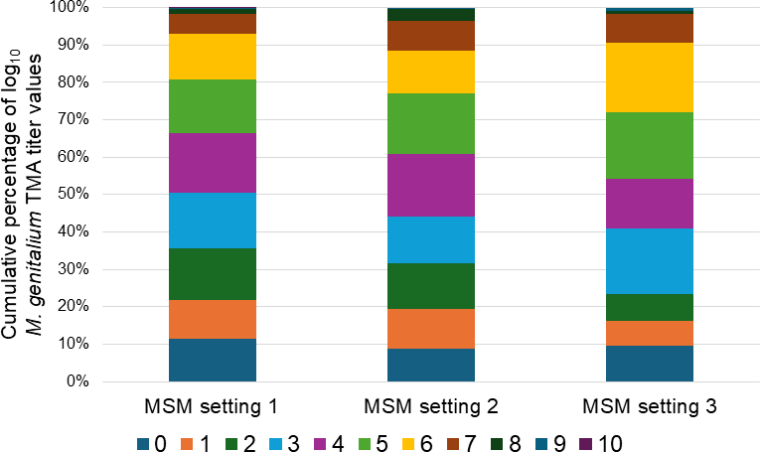
Comparative distribution of semi-quantitative *M. genitalium* RNA determination values from primary clinical specimens initially screening positive for *M. genitalium* by commercial TMA and derived from MSM settings 1, 2, and 3.

No differences in ASR detection rates (61.3%–64.9%) were noted between MSM settings (*P* ≥ 0.33; Table 2). However, both median and mean log_10_ *M. genitalium* TMA titer data from MSM setting 1 were lower than either of the other settings (*P* ≤ 0.01 for mean log_10_ titer data). In addition, approximately 25% of the log_10_ *M. genitalium* TMA titer distribution data for MSM settings 2 and 3 had values ≥6 (Fig. 2). Furthermore, >50% of MSM setting 1 log_10_ *M. genitalium* TMA titer values were ≤3.

## DISCUSSION

Several contemporaneous reports have chronicled the frequency of *M. genitalium* macrolide treatment failure and associated genetic determinant detection. Mullis *et al*. (28) reported within a US testing population that only 33% of initially *M. genitalium*-positive patients experienced microbiologic cure (determined by a nucleic acid amplification testing result of non-detected at least 21 days after initial *M. genitalium* diagnosis) upon macrolide therapy. In a university setting, Lavey *et al*. (19) reported that 30% of macrolide therapeutic regimens resulted in no *M. genitalium* RNA detection upon follow-up TMA testing at a median of 100 days after initial diagnosis. From primary clinical specimens yielding detectable *M. genitalium* nucleic acid, estimates of MRM detection have ranged from 22% to 60% in Asia (29–32), 37% to 68% in Europe (33–36), and 59% to 89% in the United States (37, 38). Data from our report, utilizing a *C_T_* cutoff value of <40, reveal an MRM rate of 57.9% in US specimens. *C_T_* cutoff-derived data have been debated in the literature (39), fall within the discretion of the LDT end-user, and may potentially underestimate the incidence of MRM within *M. genitalium*-positive primary clinical specimens (40).

In the United States, the CDC recommends a first-line, 7-day course of doxycycline therapy for *M. genitalium* infection (9). When an *M. genitalium* macrolide resistance testing result is unavailable to the provider, first-line therapy is followed by a 7-day course of moxifloxacin. In situations when an *M. genitalium* macrolide resistance testing result would be available, susceptibility (or wild-type determination) would indicate azithromycin therapeutic follow-up, while macrolide resistance would revert to moxifloxacin. Due to the paucity of cleared or available assays in the United States, these guidelines can be limiting to providers. Reports have described the development of an LDT on an automated platform that can allow for high-throughput MRM determination from primary clinical specimens (17, 41). The turnaround time for this MRM-LDT is less than 2.5 hours (including nucleic acid extraction). Moreover, the possibility for reflexive MRM determination from a *M. genitalium* TMA-positive specimen that is already housed on the analyzer exists.

Manhart et al. (37) recently characterized *M. genitalium* MRM incidence across the United States. Using a database of 286 *M*. *genitalium*-positive specimens collected from six locations, the authors noted differences in geographic distribution of MRM (though one location did not submit specimens for MRM confirmation) and in detection of MRM from females diagnosed with lower reproductive tract disease versus those who were not. The MRM detection rate from 41 MSM specimens was 51.2%, and no gender-specific differences were noted as a function of symptomatic status (*P* ≥ 0.24). The high-throughput MRM-LDT discussed in the current report, facilitating the screening of large numbers of *M. genitalium*-positive specimens, has not only provided important data on specimen source-specific MRM detection rates (42) but also provides additional demographic insight into *M. genitalium* MRM in this report.

In our database of 1,242 MRM-LDT-positive specimens, increased macrolide resistance detection was noted in the MSM (62.0% of 1,557 *M*. *genitalium* TMA-positive specimens; Table 2) and university cohorts. Such increases were elevated compared to the community-based testing population (43.2% of 458 *M*. *genitalium* TMA-positive specimens). It is noteworthy that the two community testing locations were regionally separated but noted to be within high-prevalence STI communities (23, 24). Moreover, MRM-LDT detection rates between the two community sites did not vary (*P* = 0.73). Data were available from the university settings for a symptomatic-based assessment of MRM-LDT. Nearly equal numbers of students with initial *M. genitalium* TMA-positive results were stratified into the asymptomatic and symptomatic designations (Table 3). Both MRM-LDT rate (71.4%) and mean log_10_ *M. genitalium* TMA titer data were significantly higher in symptomatic students (*P* ≤ 0.036), as compared to asymptomatic students (46.7% MRM-LDT rate). When symptomatic data were compared among participating universities, no differences in mean log_10_ *M. genitalium* TMA titer and MRM-LDT were observed (*P* ≥ 0.26). The finding of an increased MRM-LDT rate in symptomatic individuals warrants additional study to determine generalizability to other university populations or even to community care populations.

A recent publication (42) introduced the possibility of using semi-quantitative log_10_ *M. genitalium* TMA titer determination as a surrogate to adopting the MRM-LDT or to not having access to the platform. Notwithstanding the laboratory and/or clinical validation necessary to implement the surrogate test, in Table 1 of this report, log_10_ *M. genitalium* TMA titers of ≥4 were highly predictive of a positive MRM-LDT result. However, 7.3% of specimens with a log_10_ *M. genitalium* TMA titer of 1 also yielded a positive MRM-LDT result. Conversely, 5.2% of specimens with a log_10_ *M. genitalium* TMA titer of 8 yielded a negative MRM-LDT result, with this potential discordance influenced by an abundance of wild-type *M. genitalium* nucleic acid (17). Differences in log_10_ *M. genitalium* TMA titer within a demographic category further do not contribute to the prediction of MRM-LDT result. As an example, >50% of log_10_ *M. genitalium* TMA titer data from university setting 2 had a value ≤3. In contrast, >50% of log_10_ *M. genitalium* TMA titer data from university setting 1 had a value ≥4 (Fig. 1). With that said, no difference in MRM-LDT rate was realized between the two settings (*P* = 0.25). Similarly, while no difference in MRM-LDT rate was discerned between the three MSM settings (*P* ≥ 0.33; Table 2), differences in low-level log_10_ *M. genitalium* TMA titer distribution were noted between MSM setting 1 and both MSM settings 2 and 3 (Fig. 2). Taken together, these data demonstrate the necessity of defined laboratory assays such as MRM-LDT to contribute to the management of *M. genitalium* infection. However, from an epidemiologic standpoint, differences in *M. genitalium* burden (as determined by TMA semi-quantitation) within a shared demographic (i.e., MSM, university) may factor into population-based strategies for disease prevention.

Potential relevance of a purported correlation of specimens with increased log_10_ *M. genitalium* TMA titer and MRM-LDT detection may be twofold. This paradigm may suggest that macrolide-resistant *M. genitalium* exhibits more robust *in vitro* growth and/or an *in vivo* selection phenomenon in which unsuccessfully treated patients render a high-titer infection upon repeat testing. Wood et al. (43) reported similar *in vitro* culture kinetics (using genomes/mL as a reportable value) for two macrolide-susceptible and two macrolide-resistant clinical isolates of *M. genitalium*. The same group reported that 20% of a cohort with *M. genitalium* infection that was susceptible to azithromycin by way of phenotypic antimicrobial susceptibility testing, was treated with the agent, and who returned for clinical follow-up had *M. genitalium* infection that was resistant to azithromycin. While the *M. genitalium* strain types at both visits were identical, no quantitative data were reported.

A limitation of this study pertains to the acquisition of a number of these data through investigational protocols that involve screening of asymptomatic persons. Many of these protocols were designed and implemented prior to the novel (with respect to *M. genitalium*) CDC STI treatment guidelines published in 2021 (9), which do not recommend asymptomatic or extragenital screening for *M. genitalium*. While MRM detection in such settings can potentiate unnecessary utilization of macrolide agents, the value of screening for *M. genitalium*, in general, may still lie in further elucidating epidemiologic relationships with HIV and other STI agents. As an example, recent data have demonstrated an association between rectal swab *M. genitalium* detection and HIV seropositive status in MSM (44, 45). Furthermore, our study did not investigate the potential role of fluoroquinolone resistance (8, 29, 32–34, 38) in the distribution of resistant *M. genitalium* in these demographic settings.

In conclusion, data generated from a high-throughput, automated system and presented in this report expand upon knowledge of *M. genitalium*-specific macrolide resistance in the United States and may further inform providers on population- or demographic-based considerations for MRM determination in *M. genitalium*.
